# Compensating Couplant Effects in Phased-Array Ultrasonic ToF Sensing for Residual Stress

**DOI:** 10.3390/s26133975

**Published:** 2026-06-23

**Authors:** Brandon Mills, Yashar Javadi, Charles N. Macleod

**Affiliations:** 1 Department of Electrical and Electronic Engineering, University of Strathclyde, Glasgow G1 1XQ, UK; 2 Department of Design, Manufacture, and Engineering Management, University of Strathclyde, Glasgow G1 1XQ, UK

**Keywords:** residual stress, phased array ultrasonic transducers, acoustic simulation, non-destructive testing

## Abstract

**Highlights:**

**What are the main findings?**

- Couplant thickness significantly biases LCR time-of-flight measurements, causing residual stress errors of ~36 MPa (~13% of yield strength) if uncorrected.
- A model-informed compensation workflow reduced ToF bias to ~0.3 ns in experiments, lowering residual stress error to ~1.1 MPa (~0.4% of yield strength).

**What are the implications of the main findings?**

- Provides practical calibration and uncertainty-quantification guidance for phased-array ultrasonic residual stress sensing.
- Establishes a foundation for further validation towards field deployment and in-process monitoring of residual stress and microstructure evolution in welding and additive manufacturing.

**Abstract:**

Residual stress (RS) is a key integrity parameter after welding and additive manufacturing, motivating portable sensing methods for in-situ assessment. Phased Array Ultrasonics for Residual Stress (PAURS) treats a phased-array probe as a time-of-flight (ToF) sensor and infers RS from ToF changes of the longitudinal critically refracted (LCR) wave propagating near the surface. In practical deployments, however, the ToF sensing chain can be susceptible to systematic bias from sensor–specimen interface variability (couplant layer thickness) which can dominate the inferred stress uncertainty if not quantified and corrected. This study combines numerical modelling with experimental validation to (i) characterise couplant-induced sensitivity in LCR ToF sensing, (ii) propagate this effect into RS error/uncertainty, and (iii) demonstrate a model-informed compensation strategy suitable for practical calibration workflows. Simulations show that couplant thickness variations can introduce RS errors of ~36 MPa (~13% of yield strength). The proposed compensation reduces ToF bias to 0 ns under idealised simulated conditions and to ~0.3 ns in experiments, corresponding to ~1.1 MPa RS error (~0.4% of yield strength). These results provide configuration-specific guidance for sensor calibration and uncertainty reporting in phased-array ultrasonic RS sensing, and establish a foundation for future in-process sensing of residual stress and microstructure evolution.

## 1. Introduction

Longitudinal critically refracted (LCR) waves are an informative tool for measuring residual stresses in metallic components [1,2]. These methods rely on detecting small variations in ultrasonic Time-of-Flight (ToF), which are correlated with elastic property changes induced by residual stress fields due to the acoustoelastic effect [3]. The precision of such measurements is crucial, especially in applications involving fatigue-critical parts such as in aerospace or nuclear industries [4,5].

**Figure 1:**
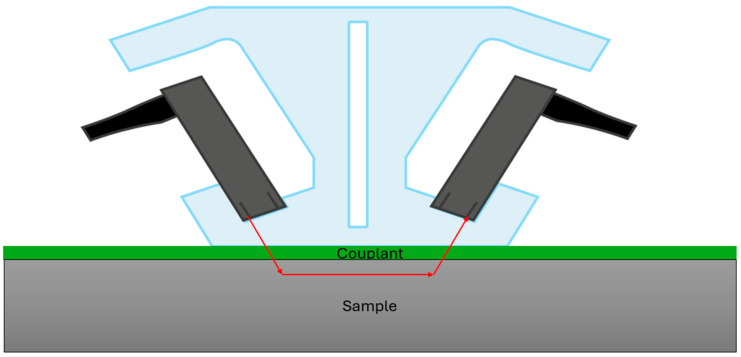
Schematic design of experimental setup, with an example acoustic path marked in red.

The LCR wave in the Phased Array Ultrasonics for Residual Stress Measurement (PAURS) inspection process is generated using a specialised wedge following the methodology set out by Mills et al. [2] (Figure 1). The wedge ensures that the wave enters the sample under test at the critical angle, allowing propagation parallel to the surface. This wave is chosen as its speed of sound is the most sensitive to changes in material stress [6].

However, various aspects of the inspection process can induce an error in the expected results. It has been shown that a ToF difference of 1 ns is equivalent to roughly 4 MPa of Stress [7]. This small margin can be erroneously observed through small changes in the ToF due to sample temperature, couplant thickness, and wedge tilt. Certain mitigations can be taken, such as considering a second echo as a reference point in pulse-echo configurations [7] or, for pitch-catch probe setups, consider a ToF difference between receivers. The receiver difference compensation method is best matched to the pitch catch setup used in this work.

This paper presents a simulation-based study of gel thickness effects on ultrasonic LCR wave propagation, specifically focusing on time-of-flight variations. Simulations were performed here using a numerical modelling approach to simulate ultrasonic wave propagation through a multilayer system consisting of the transducer, coupling gel, and test material using the KWave MATLAB toolbox in MATLAB 2024a, which has previously been used for ultrasonic simulation [8,9,10,11]. A parametric analysis was conducted by varying the gel thickness while keeping material properties and wave incidence constant. The simulations provided insight into both the magnitude and nature of ToF shifts associated with realistic gel thickness variations.

Several previous studies have investigated the influence of couplant layers and surface conditions on ultrasonic wave propagation and time-of-flight measurements. Solid theoretical groundings have been provided by Zverev [12] and Haslinger [13,14]. Further experimental work by Kim et al. [15] showed that variability in couplant thickness can cause measurable shifts in amplitude. From a modelling perspective, research Leymarie et al. [16] emphasized the need for high-fidelity simulation tools to capture complex wave interactions across layered interfaces, especially when small timing differences are critical. Finite Element Modelling approaches, such as those by Vijigiri et al. [17] and Kogut [18] have been used to assess wave sensitivity to contact conditions.

This work contributes to the understanding of systematic uncertainties in ultrasonic residual stress measurements and proposes an approach to quantify and compensate for the gel-induced error within the specific PAURS pitch-catch FMC configuration used for LCR-based residual stress inspections. Such refinements are essential to increase confidence in ultrasonic approaches for residual stress, particularly when applying these techniques outside controlled laboratory environments. The findings here are specific to the tested probe, wedge, and sample conditions, and further validation is required before generalisation.

In this study, calibration refers to the conversion between measured ToF change and residual stress equivalent error using the applied acoustoelastic approximation. Compensation refers to the adjacent-receiver subtraction procedure used to reduce the common couplant induced timing bias. Uncertainty quantification refers to the estimation and reporting of the remaining ToF and stress-equivalent variation after compensation. The proposed procedure is used primarily as a compensation method, with direct relevance to calibration and uncertainty reporting for PAURS measurements.

## 2. Theory

The ultrasonic methodology for residual stress measurement relies on detecting small changes in the material speed of sound, based on the principle of acoustoelasticity. This states that material sound velocity is dependent on the material stress, and can be expressed with Equation (1) below [19]:

(1) $$L=-\frac{2\lambda \left(\lambda +2\mu \right)+\lambda A+2\left(\lambda -\mu \right)B-2\mu C}{\lambda +\mu }$$

where L is the acoustoelastic constant, A, B, and C are the third order Landau constants [20], and λ and μ are the second order Lamé constants, given by:

(2) $$\lambda =\frac{E\nu }{\left(1+\nu \right)\left(1-2\nu \right)}$$

(3) $$\mu =\frac{E}{2\left(1+\nu \right)}$$

where E is the Young’s Modulus and ν is the Poisson Ratio. This value is then used to obtain the residual stress using:

(4) $$\Delta \sigma =-\frac{E\cdot dt}{L\cdot t_{0}}$$

where Δσ is the residual stress, $t_{0}$ is the Time of Flight of the ultrasonic wave in an unstressed material, and dt the difference in Time of Flight between a stressed and unstressed area. This dt value is the main focus of this study. In typical LCR residual stress measurement, the dt values are on the order of nanoseconds, with a standard structural steel weld with no stress reduction peaking around 300 MPa, and a yield strength of 350 MPa [2]. Therefore, this difference can be obscured by other factors, such as sample temperature or couplant gel thickness. Due to a given material’s average surface roughness, the gel thickness can never be considered consistent. This can cause minor deflections in the acoustic path that alters the Time of Flight, and therefore the measured stress. Standard practice in both Phased Array and Single Element pitch-catch LCR setups is to reduce the gel coupling thickness effect by subtracting the Time of Flight between two adjacent receivers.

## 3. Materials and Methods

### 3.1. Simulation

**Figure 2:**
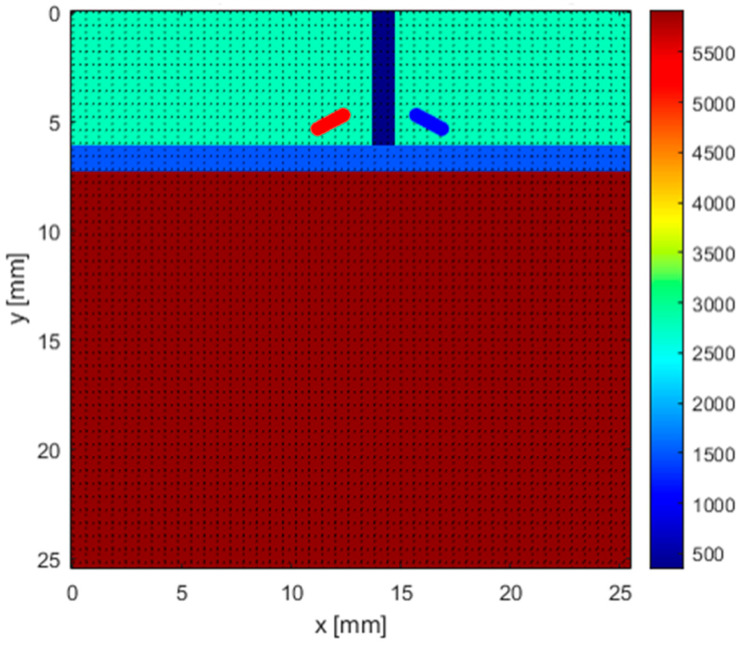
Two-dimensional k-Wave mesh representing a cross-section through the pitch-catch inspection geometry, presented as a speed of sound map.

The KWave toolbox in MATLAB 2024a was utilised along with a simplified mesh (Figure 2) consisting of a wedge, sample, airgap, and a layer of acoustic couplant gel. The 0.2 mm spatial step was selected as a compromise between computational cost and the need to represent the full configuration under testing across a large parametric sweep. A formal mesh-convergence study was outside the scope of this work. Consequently, the model must be interpreted as providing comparative trends in ToF behaviour, and the experimental validation used to establish the practical performance of the proposed compensation approach. The discretisation limits the precision that thin couplant layers can be represented with, particularly as the couplant thickness approaches the scale of the grid spacing. The simulation results are here interpreted as configuration-specific comparative ToF trends. The number of nodes in both the x and y directions were set to 128 for the same reasons. The speed of sound of the wedge, couplant, and material were set to 2788 ms^−1^, 1500 ms^−1^, and 5920 ms^−1^, respectively, taken from existing datasheets, and air set to the standard of 343 ms^−1^. The simulations were performed using the acoustic time-domain formulation in k-Wave. The model did not explicitly simulate mode-conversion, transducer dynamics, or detailed contact mechanics, and so is considered a comparative timing model.

The model used the standard k-Wave absorbing boundary layer implementation to reduce reflections from the computational boundaries. Attenuation and dispersion were not included, so the model isolates the effect of geometry and couplant thickness variation on ToF rather than attenuation dependent amplitude change. The influence of couplant viscosity and contact mechanics were not explicitly represented.

The model is a 2D cross-sectional depiction of the experimental setup. This approximation was selected in order to reduce computational cost and to isolate the timing influence of the couplant thickness variation.

**Figure 3:**
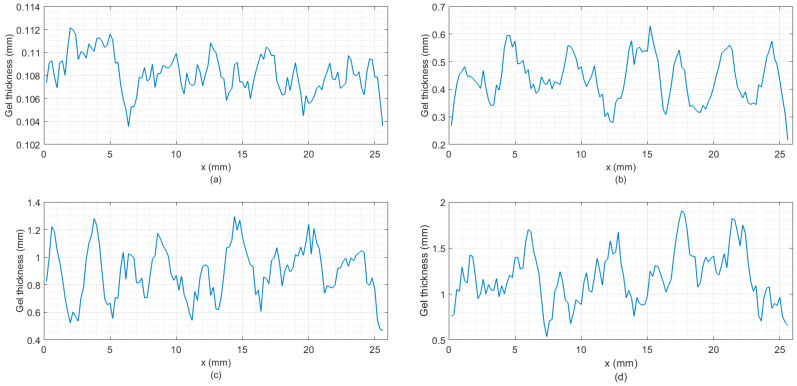
A selection of gel thickness profiles at (**a**) iteration 1 (**b**) iteration 41, (**c**) iteration 101, and (**d**) iteration 141.

Additionally, two ultrasonic transducers were simulated, each with eight elements, a frequency of 5 MHz, and a pitch of 0.5 mm to match those used for experimental validation. The array elements were defined through source and sensor masks corresponding to the nominal element positions, and so the transducer backing and piezoelectric material response were not explicitly represented. These were set up in pitch-catch configuration, with an angle of 28.1° to induce an LCR wave. This setup produces an LCR wave with a penetration depth of approximately 2.6 mm, from the formula given in Mills et al. [2]:

(5) $$D=cf^{-0.95}$$

where D is the penetration depth, c the speed of sound in the material, and f the frequency. The script was run using an Nvidia RTX 3050 GPU to decrease computation time and maximise the number of data points obtainable in each simulation run. The data was collected in the Full Matrix Capture (FMC) format, such that every individual transmitting element was received at each receiving element, as opposed to a linear scan where each transmitting element is only received by its opposite receiving element (i.e., T1-R1, T2-R2, etc.). Configuring the scans in this way allowed for squaring the number of data points per iteration, essential for the planned gel compensation methodology. Additionally, due to real world material properties, the surface of the sample was considered to have an inherent roughness, and so the gel thickness was expected to vary from point to point along the wedge and gel interface. To model this, an array of 128 random numbers (to match the number of nodes in the x direction of the simulation) between 0 and 1 and smoothed through convolution was applied to Equation (6) below:

(6) $$\mathrm{Thickness}\;\mathrm{Profile}=t_{min}+\left(t_{max}-t_{min}\right)\cdot \left(x_{n}/n\right)\cdot \mathrm{Noise}\;\mathrm{Profile}$$

where $t_{max}\;\mathrm{and}\;t_{min}$ are the maximum and minimum thicknesses, *n* the number of iterations, $x_{n}$ the particular iteration of note, and the noise profile. To fit the output thickness profile to the mesh, it was divided by the mesh separation value of 200 µm. This produced a gel thickness that varied across a small number of values, that gradually increased with each iteration, some examples of which are shown in Figure 3:

The simulated couplant profiles were selected to cover both realistic roughness-scale variability and deliberately excessive wedge-sample separation. Outside controlled conditions, typical gel thickness will vary based on the surface roughness of an object, and so the minimum can vary from 0.025 µm to 50 µm [21]. The larger sub-millimetre to millimetre scale values are used here to represent poor probe seating, wedge tilt, or visible separation. These larger values were included to identify the approximate region where a valid LCR path is lost and the measured ToF no longer represents propagation in the sample. The results below demonstrate how couplant thickness variation influences ultrasonic propagation and, consequently, residual stress calculation.

### 3.2. Experimental Verification

**Figure 4:**
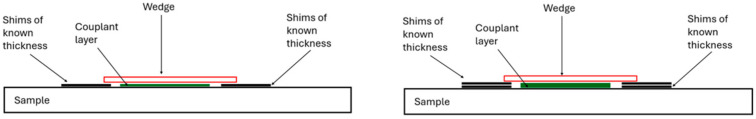
Schematic diagram of increasing gel thickness in experimental setup.

In order to validate these simulations, an experimental setup was designed to allow for a known gel thickness between the sample under test and the wedge. Steel shims of 0.1 mm thickness were placed on the sample to impose a nominal wedge-sample separation, and additional shims were layered to increase this separation (Figure 4). The local couplant thickness was not independently measured across the aperture, and the reported thickness values refer to this nominal separation. Though local compression and surface roughness may have caused the actual couplant layer to vary spatially, it was assumed that the mean gel thickness across the wedge aperture would be equal to the nominal separation.

**Figure 5:**
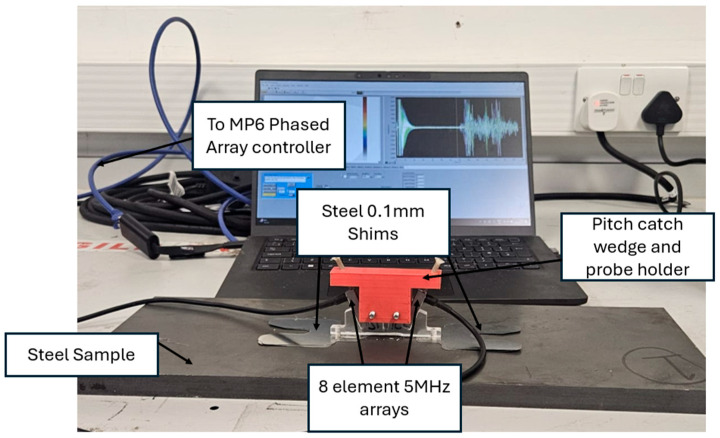
Annotated experimental setup.

The experimental setup is shown below in Figure 5. Three identical rolled samples of S275 structural steel were used, with consistent, low residual stress values expected. A PEAK MP6 microcontroller (PEAK NDT, Derby, UK) was used to drive the two 5 MHz, 8 element, 0.5 mm pitch Olympus transducers (Evident, Hamburg, Germany) with a 100 MHz sampling frequency, placed into a custom Rexolite (Sourced from Stockline Plastics, Glssgow, UK) wedge with a 28.1° probe angle for LCR wave inducement. The shims used were 0.1 mm thick steel. From Equation (5), it was determined that when couplant thickness approached 0.8 mm, all the LCR wave energy would be contained within the couplant as opposed to the sample, and therefore a valid ToF from within the sample would not be obtained. To account for this, gel thickness was only considered up to 0.7 mm experimentally.

Each shim-defined separation condition was acquired once per S275 plate, giving three plate-level repetitions for each nominal separation. The mean and variation across the three plates and 64 initial acoustic paths are reported in the results section. Measurements were performed under laboratory conditions at approximately 24 °C. Probe pressure was applied manually using the wedge and probe holder, and was kept nominally consistent between measurements, but was not independently measured. The couplant used was produced by Diagnostic Sonar Ltd., Edinburgh, UK; its viscosity was not characterised during the experiments. Probe positioning was maintained within the wedge using the holder shown in Figure 5.

On every transmitter–receiver path, the LCR arrival was identified within a fixed time gate. The ToF of the wave was then extracted from the second zero crossing of the LCR wave within this gate, to ensure a consistent reference point across all scans. This was applied to both experimental and simulated waveforms. This resulting ToF from each transmitter–receiver pair was then subtracted across adjacent paths, and these compensated ToF values were subtracted from that of the perfectly coupled reference case.

## 4. Results

### 4.1. Simulation

**Figure 6:**
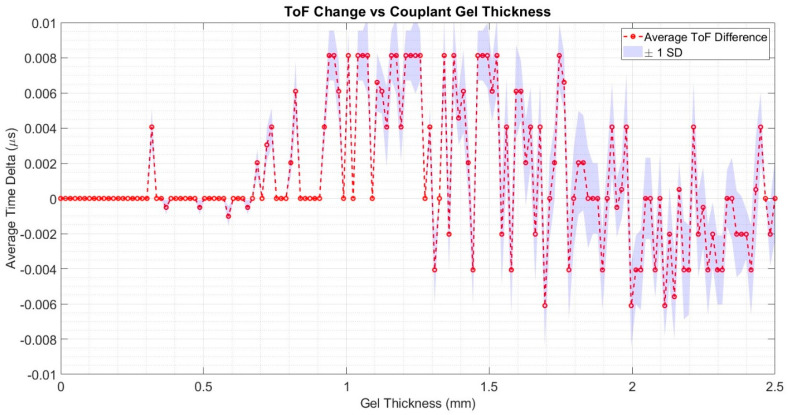
Graph of average gel thickness against change in ToF from ideal scenario, from 0 mm to 2.5 mm.

As discussed in the methodology section, the gel thickness was modelled as a varying profile, to better represent the surface roughness characteristics of a standard sample. As surface roughness varies spatially, the gel thickness will also vary as different surface depths can accommodate different volumes of gel. In Figure 6, the average gel thickness is plotted against the average change in ToF from ideal conditions.

A zero-crossing ToF picking method was used here, and the second zero crossing selected as a consistent reference point, as it is less directly affected by amplitude variation than the maximum peak value. The method remains sensitive to distortion and changes in signal morphology due to poor coupling. For this reason, the same time gate and zero-crossing reference were applied consistently across all cases, and the resulting values are interpreted as comparative ToF changes. Cross-correlation and phase-based methods may offer improved robustness where waveform shape remains consistent, but can become unreliable where the waveform morphology changes, as it does in this study. A comparison of these ToF-picking methods is therefore identified as future work.

**Figure 7:**
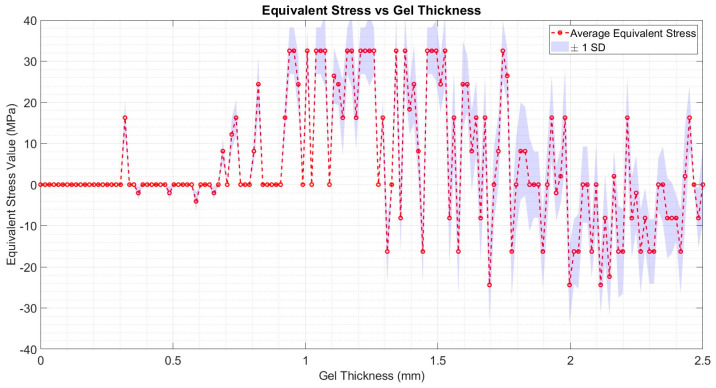
Graph of average gel thickness against approximate stress error from ideal scenario, from 0 mm to 2.5 mm.

It was noted above that in steel, the LCR penetration depth is 2.6 mm, while the maximum LCR depth in couplant is around 0.8 mm. As the couplant layer approaches this scale, the received signal can no longer be considered as a valid steel sub-surface LCR measurement, and so the increased ToF variability beyond this region is treated as a loss of the intended LCR path within the sample. At larger separations, the received wave demonstrates couplant dominated propagation and altered refraction at the interfaces. These results are useful for identifying a practical failure regime, but any stress-equivalent values calculated from this region cannot be treated as physically meaningful residual stress values, though they have the potential to be misinterpreted as such. The equivalent stress using the approximation described above is presented in Figure 7:

Though the overall maximum stress error is low, it is important to note that this sample is considered to have minimal residual stress, and the scans have occurred in the same position upon the sample. Therefore, the 36 MPa equivalent stress error is considered to be a potential uncertainty present in a standard inspection. This error is 13% of the yield strength of the sample and, if this were to be unconsidered in a true residual stress inspection, could result in a dangerous underreporting of the present residual stress.

As previously mentioned, standard practice in Single Element ultrasonic residual stress measurement systems is to take the difference in Time of Flight between two receivers as the ToF to reduce the gel factor. Here, using PAURS, one has the opportunity to take multiple subtractions per transmitter. For example, in this eight-element setup, if only adjacent receiving elements are considered for subtraction, seven values per transmitter are obtained, or 56 total—enough for a robust average value. This is described in Equation (7) below:

(7) $$\Delta t=\frac{\sum_{i=1}^{n}\sum_{j=1}^{n-1}t_{i,j+1}-t_{i,j}}{n}$$

**Figure 8:**
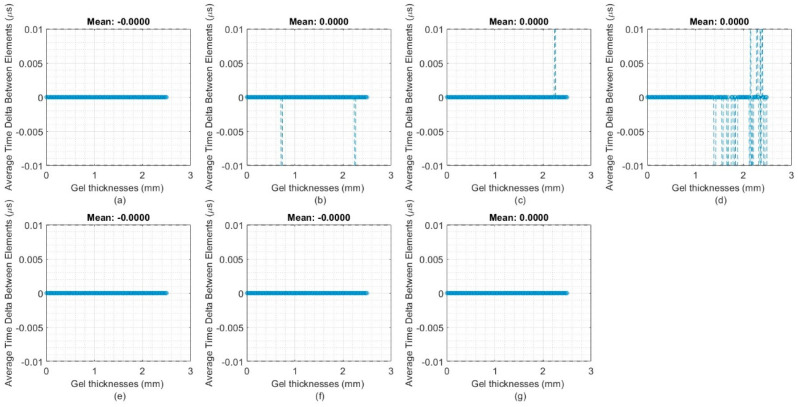
An example of per element gel compensated results. (**a**) Rx2-Rx1, (**b**) Rx3-Rx2, (**c**) Rx4-Rx3, (**d**) Rx5-Rx4, (**e**) Rx6-Rx5, (**f**) Rx7-Rx6, (**g**) Rx8-Rx7.

Here, $t_{i,j}$ is the measured ToF from transmitting element i to receiving element j, while $t_{i,j+1}$ is the measured ToF from the same transmitting element to the adjacent receiving element. Additionally, n denotes the total number of elements within a transducer, and Δt the mean inter-path time difference. Due to the nature of this compensation method, a total of 56 path-wise differences is obtained and averaged from the full 8 × 8 element FMC dataset, an example of which is shown in Figure 8:

It can be seen here that barring few outliers in receiver pairs 3-2, 4-3, and 5-4, the difference in time is consistently 0 µs, which is to be expected, as there is no stress effect on the Time of Flight.

**Figure 9:**
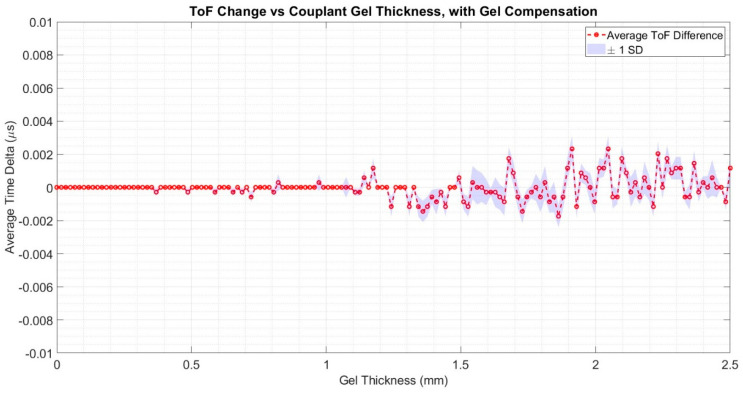
Average ToF difference when per element subtraction is implemented.

**Figure 10:**
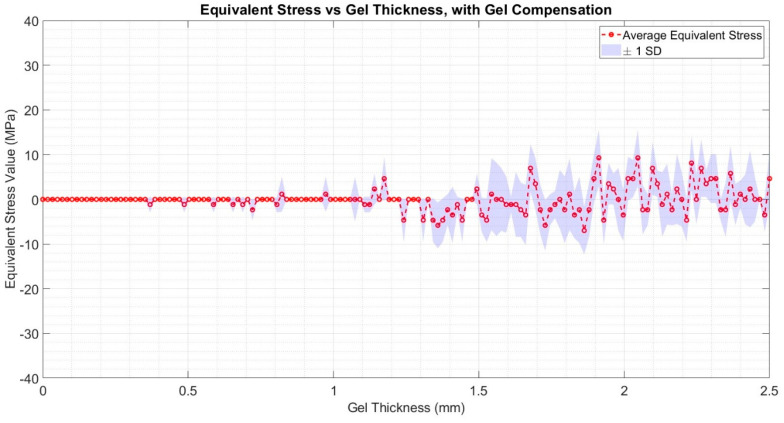
Graph of average gel thickness against approximate stress error from ideal scenario, from 0 mm to 2.5 mm, when per element subtraction is implemented.

When all 56 subtractions are averaged, an overall mean of 0 ns is obtained, and although results are still erratic after a point, that point has been pushed back by 0.3 mm of gel thickness to around 1.1 mm, as described by Figure 9:

This is also demonstrated when the equivalent stress is considered in Figure 10, which also shows much smaller and consistent values than in the uncompensated case.

Here, with a peak of 2 ns of Time of Flight variation and around 10 MPa of equivalent stress, a significant reduction in error can be clearly observed. The high error region begins after 1.1 mm of gel thickness, corresponding to a wedge-sample separation that is visible to the naked eye. Under the tested conditions, the adjacent-receiver subtraction method substantially reduced the couplant-induced timing error. This method cannot be interpreted as fully eliminating this couplant effect, as coupling, wedge, and probe alignment uncertainties remain under physical testing. As discussed in the methodology section, the sudden and erratic behaviour at larger gel thickness values is associated with the ultrasonic pulse no longer following a stable LCR path within the sample.

These results are supported by experimental validation below, where in order to ensure that only the gel’s effect on the Time of Flight within the sample under test is considered, gel thickness to a maximum of 0.7 mm was considered.

### 4.2. Experimental Validation

**Figure 11:**
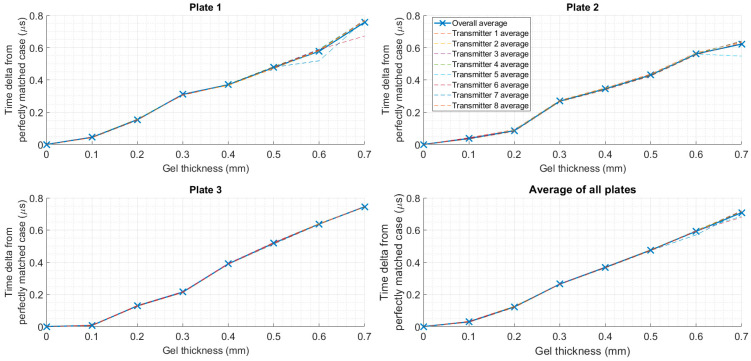
Graphs of increases in the ToF from perfectly matched case due to increases in gel thickness. The legend indicated here applies also to Figure 12 and Figure 13.

Experimental validation followed similar trends to the simulated results. As mentioned above, the experimental validation focused on the gel thickness case, as that showed the greatest variability. Initial analysis showed that the time delta from the perfectly matched 0 mm case increased linearly with increases in gel thickness (Figure 11).

The simulated and experimental error magnitudes represent different couplant thickness conditions and cannot be interpreted as directly equivalent. The simulated stress value of 36 MPa corresponds to the stress-equivalent error predicted by the idealised k-Wave model over the imposed couplant thickness profile. In contrast, the larger uncompensated values found experimentally arise from the deliberate shim defined wedge-sample separations, which are used as a controlled sensitivity test and failure-envelope assessment. The compensated values are the main basis for assessing the proposed adjacent-receiver correction methodology.

**Figure 12:**
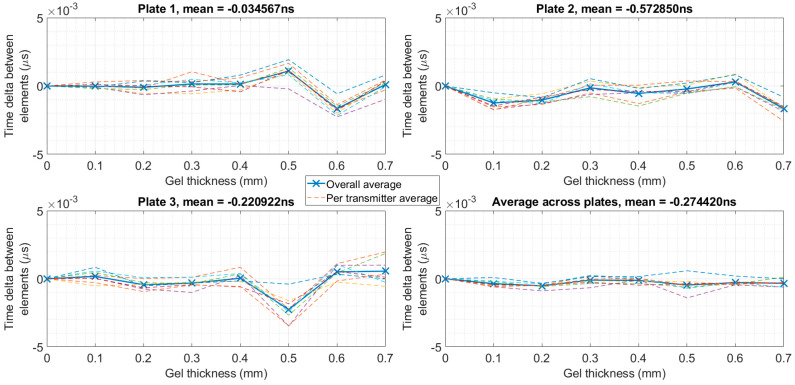
Graphs of changes in the ToF from perfectly matched case due to increases in gel thickness.

As seen above in Figure 6, at low gel thicknesses, the deviation between the mean delta and the per element delta is comparatively low, even as the time delta from the perfectly matched case grows. As in Section 4.1, the subtraction methodology of Equation (7) was applied to the experimental data in order to compensate for the gel thickness, as even the minimal difference here of 0.04 µs at 0.1 mm of gel thickness is equivalent to 160 MPa of stress error, as per the general rule mentioned above. When considering the 0.7 µs difference at 0.7 mm, the need for compensation becomes very clear. When the compensation method was applied, the average ToF reduced to 0.3 ns across all three plates, with a maximum value of around 0.57 ns and a minimum of 0.03 ns (Figure 12).

**Figure 13:**
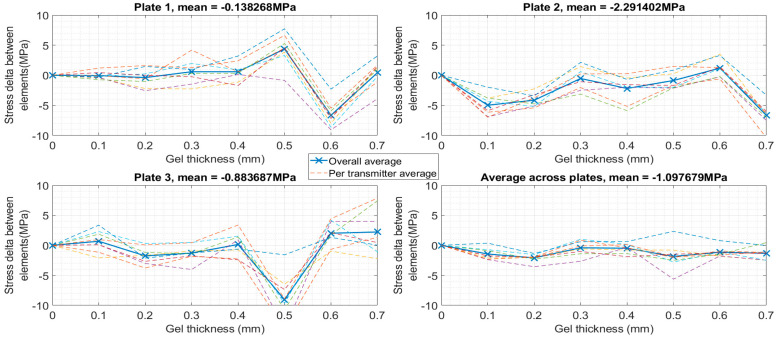
Stress approximation graphs showing the difference from perfectly matched case due to increases in gel thickness.

This compensation technique substantially reduced the effect of acoustic couplant thickness on the residual stress equivalent error. Applying the 1 ns ≈ 4 MPa approximation, the average ToF error of 0.27 ns corresponds to approximately 1.1 MPa of residual stress error. This indicates that the adjacent-receiver subtraction method strongly reduced the couplant induced timing error under the conditions tested here. However, this did not fully remove all sources of timing related error. This stress approximation is shown in Figure 13.

These results are significant, as they indicate that so long as the LCR wave can be detected on a good number of adjacent elements, even a significant separation between the sample and the wedge of 0.7 mm can be compensated for, and so any µm scale variations in gel thickness due to surface roughness can also be computationally calibrated for to a reasonable degree, though the effects are not assumed to be fully removed. The simulated and experimental results cannot be treated as a one-to-one comparison as the simulation used an idealised acoustic model, while the experiments included real contact effects, small variations in probe tilt or force, and resultant gel displacement effects. The relevant comparison is therefore the dominant trend: that increasing couplant separation produced larger uncompensated timing shifts, while application of the adjacent receiver-subtraction method substantially reduced the timing error. The remaining experimental residual timing bias is attributed to interface and acquisition effects not present in the simplified model.

The stress equivalent error values reported here should be interpreted as the apparent stress error associated with measured ToF bias. The remaining uncertainty sources include surface roughness, local couplant redistribution, probe pressure, wedge seating, and probe positioning. In this study, the adjacent-receiver subtraction compensation method is used to reduce only the effects of couplant on the acoustic ToF, and the remaining uncertainty terms are treated as limitations requiring further investigation. A full uncertainty budget, partitioning the contributions of individual factors, was beyond the scope of this study and remains an important area for future investigations.

## 5. Conclusions

The study investigated the influence of couplant-thickness variation on PAURS-based LCR ToF measurements using k-Wave acoustic simulation and controlled laboratory experiments. The simulations demonstrated that increasing couplant thickness can introduce apparent ToF shifts that correspond to residual stress equivalent errors of up to approximately 36 MPa in the tested configuration. The experimental shim validation study confirmed that this imposed couplant separation can produce large uncompensated timing shift, demonstrating the practical need for a compensation technique.

Adjacent-receiver subtraction substantially reduced the couplant induced ToF bias. In simulation, the average compensated ToF difference was reduced to approximately 0 ns under idealised numerical model conditions. In the experimental validation, the average ToF error reduced to approximately 0.27–0.3 ns, corresponding to an equivalent residual stress error of 1.1 MPa using the conversion factor applied in this study. These results show that the proposed compensation procedure can reduce this couplant-induced bias under the tested probe and specimen configuration, although it does not remove all coupling or acquisition related uncertainty. Future work will consist of assessing this validation method across additional probe and specimen configurations, as well as applying alternative methods for identifying the zero-crossing reference point.

## Data Availability

Dataset available on request from the authors.

## Abbreviations

The following abbreviations are used in this manuscript:

RS	Residual Stress
PAURS	Phased Array Ultrasonics for Residual Stress Measurement
LCR	Longitudinal Critical Refraction
ToF	Time of Flight
